# Pericyte–Glioblastoma Cell Interaction: A Key Target to Prevent Glioblastoma Progression

**DOI:** 10.3390/cells12091324

**Published:** 2023-05-05

**Authors:** Ana Pombero, Raquel Garcia-Lopez, Salvador Martínez

**Affiliations:** 1 Instituto de Neurociencias, Universidad Miguel Hernández–CSIC, Campus de San Juan, Avda. Ramón y Cajal sn, 03550 Alicante, Spain; apombero@umh.es (A.P.); r.garlo@umh.es (R.G.-L.); 2 Centro de Investigación Biomédica en Red en Salud Mental, CIBERSAM-ISCIII, 46010 Valencia, Spain

**Keywords:** cell–cell contact, filopodia, pericytes, glioblastoma, high-grade glial neoplasm

## Abstract

Multiple biological processes rely on direct intercellular interactions to regulate cell proliferation and migration in embryonic development and cancer processes. Tumor development and growth depends on close interactions between cancer cells and cells in the tumor microenvironment. During embryonic development, morphogenetic signals and direct cell contacts control cell proliferation, polarity, and morphogenesis. Cancer cells communicate with cells in the tumor niche through molecular signals and intercellular contacts, thereby modifying the vascular architecture and antitumor surveillance processes and consequently enabling tumor growth and survival. While looking for cell-to-cell signaling mechanisms that are common to both brain development and cancer progression, we have studied the infiltration process in glioblastoma multiforme (GBM), which is the most malignant primary brain tumor and with the worst prognosis. Cell-to-cell contacts, by means of filopodia-like structures, between GBM cells and brain pericytes (PCs) are necessary for adequate cell signaling during cancer infiltration; similarly, contacts between embryonic regions, via cytonemes, are required for embryo regionalization and development. This GBM–PC interaction provokes two important changes in the physiological function of these perivascular cells, namely, (i) vascular co-option with changes in cell contractility and vascular malformation, and (ii) changes in the PC transcriptome, modifying the microvesicles and protein secretome, which leads to the development of an immunosuppressive phenotype that promotes tumor immune tolerance. Moreover, the GTPase Cdc42 regulates cell polarity across organisms, from yeast to humans, playing a central role in GBM cell–PC interaction and maintaining vascular co-option. As such, a review of the molecular and cellular mechanisms underlying the development and maintenance of the physical interactions between cancer cells and PCs is of particular interest.

## 1. Introduction

Intercellular communication is a key process in cell decision-making during the development and progression of cancer. The microenvironment is regulated by cell signals that influence other cells through paracrine mechanisms, facilitating cancer cell proliferation and migration. Moreover, cell-to-cell contact is necessary to polarize cells and regulate morphogenesis and cell motility. Here, we aim to review the developmental mechanisms of intercellular communication which are reproduced in glioblastoma multiforme infiltration, resulting in vascular co-option and immune system conditioning.

Gliomas are glial tumors of the central nervous system. They are classified as: oligodendrogliomas, astrocytomas, and glioblastomas. Glioblastoma multiforme (GBM) is a high-grade infiltrative neoplasm, it is the most aggressive of all brain cancers with a low life expectancy of no more than 15 months after diagnosis [1,2]. The poor prognosis for GBM is due to its highly invasive capacity, diffuse cell organization, and infiltration capacity. Sadly, GBM is a relatively common brain tumor with an incidence of 5–7 cases per 100,000 individuals [3]. Vascular involvement in tumor progression is one of the most important characteristics of GBM, as it causes alterations to blood vessels [4] and the tumor can migrate along vessel walls in a process called vascular co-option [5]. Since the brain is a highly vascularized structure, the GBM cells’ angiotropism favors tumor expansion. Thus, infiltrating tumor cells can easily come into contact with blood vessels and obtain oxygen and nutrients without having to activate angiogenesis. Furthermore, tumor cells use preexisting vasculature as scaffolding to migrate into the stroma between vessels [6]. Co-option is a tumor cell migration process mediated by physical contacts between GBM cells and endothelial cells, extracellular matrix, or pericytes (PCs) [7,8].

Caspani et al. (2014) demonstrated that GBM cells target the PC cells in the vascular wall and are necessary for cancer infiltration into the edge of the tumor mass and tumor survival [8]. Pericytes are peri-endothelial vascular mural cells [9] located in the external/abluminal wall between the vascular feet of astrocytes and the endothelial basal membrane of small blood vessels (precapillary arterioles, capillaries, and postcapillary venules). Furthermore, PCs have been classed as mesenchymal stem cells (MSCs) through the expression of molecular markers and differentiation properties and attributed to other functions, such as angiogenesis, the synthesis of bioactive molecules related to immune response, and vascular tone/blood flow regulation [10,11]. It has been suggested that damaged or inflamed PCs become activated MSCs, producing molecules that control the immune response [12].

PCs form perivascular layers that support the vasculature and participate in the structure and function of the blood–brain barrier (BBB). Adherens junctions of endothelial cells and PC coverage are responsible for the correct function of the BBB. In fact, if pericyte coverage is lost or damaged, the BBB is compromised as its permeability increases, resulting in an accumulation of plasma-derived proteins in the extracellular space and neuronal inflammation [13]. PCs, therefore, form a fundamental part of the neurovascular unit (NVU), that is, a functional structure composed of PCs, endothelial cells, astrocytes, and neurons. The NVU describes the relationship between neural cells and their blood vessels, and it controls brain homeostasis and cerebral blood flow [10,13,14,15]. Pericytes also act as one of the brain’s structural components, where their main function is to provide the ideal environment for neural proliferation in the adult brain, which is known as the neurovascular niche (NVN). The NVN is composed of adult neural progenitors, astrocytes, endothelial cells, PCs, and extracellular matrix components [10,16,17].

The perivascular location of PCs in the Virchow–Robin space, where they are in contact with the cerebrospinal fluid and vascular feet of astrocytes, means they are perfectly positioned to control several aspects of the CNS immune response. Pericytes constitute a new class of cell-mediating immunological defense in the brain [18,19]. Furthermore, PCs express the appropriate receptors to respond to several types of inflammatory insults. Brain PCs have many properties of immunocompetent cells, expressing and responding to cytokines and co-stimulatory molecules, presenting antigen to T cells, and displaying phagocytic ability [20,21,22,23]. Pericytes express several chemokines that regulate leukocyte recruitment in response to inflammatory mediators. They also secrete inflammatory mediators that can polarize parenchymal microglia cells to either a pro- or anti-inflammatory phenotype. In fact, in vitro studies have shown that PCs secrete proinflammatory mediators following immunological activation, including IL-1b, TNF-α, IFNg, and IL-6, which can induce a proinflammatory state in astrocytes, microglia, and endothelial cells and precipitate apoptotic neuronal death [21,24,25]. Conversely, PCs can also secrete several factors involved in anti-inflammatory roles, including CX3CL1 and IL-33 [26,27].

In addition to contributing to innate immunity, PCs may also modulate adaptive CNS immune functions. Pericytes express major histocompatibility complex (MHC) class II molecules, which are type I membrane glycoproteins that bind peptide fragments derived from exogenous protein sources, including viral and bacterial pathogens, and transport them to the cell surface for recognition by helper T cells. All of this suggests that pericytes can present antigens to T cells [23,28]. Finally, several studies have shown that PCs can also regulate the expression of cytokines, chemokines, and proteases in the tumor cell niche, which may promote immunosuppression, tumor angiogenesis, growth, and metastasis [29,30,31,32].

Glioblastoma cells use PC contacts to exploit and migrate along preexisting blood vessels [5,8]. They can also regulate the pericytes’ immune properties to evade the immune response [8,33]. As such, we can learn a lot by reviewing the molecular and cell mechanisms that underlie GBM cell polarity and which help establish and maintain physical interactions between GBM cells and PCs, as well as the resulting impact on PC physiology. Indeed, recent reviews have summarized the crosstalk between GBM cells and the tumor microenvironment [34,35], but they did not delve into the physical contacts between GBM cells and PCs and their consequences on PC behavior.

## 2. Role of Vascular Co-Option in Vascular Malformation and Changes in Pericyte Contractility

### 2.1. Vascular Co-Option

It has been suggested that direct interaction between proliferating neural progenitors and vascular cells is the fundamental process behind neurogenesis and cell migration in CNS development and adult neurogenesis [36,37]. In the subventricular zone of the developing hippocampus, molecular signals from progenitors and vascular cells are both required to regulate neural proliferation and migration and to establish adequate growth of vascular niches [38]. The perivascular accumulation of neurons and neural precursors has been observed in migration streams in the developing cortex, which suggests that intercellular communication involves a co-option-like process during brain development [39]. Tsay et al. (2016) also reported that neural cells undergo perivascular migration during oligodendrocyte progenitor migration [40].

Most tumors induce angiogenesis in order to grow. However, some cover their developmental needs using preexisting vessels, which they can also migrate along, as occurs in vascular co-option [41]. Co-option was first described in metastatic lung cancer and gliomas [7], but it has also been observed in melanomas and breast, colorectal, and liver cancer [42,43,44].

The infiltration of GBM cells into surrounding tissues is a key factor in tumor recurrence. Tumor cells often reoccur within a 2–3 cm radius around the primary tumor [45,46] because of the GBM cells’ high infiltration capacity. Tumor cells use different strategies to colonize tissues, such as individual or collective migration through the extracellular matrix, perineuronal satellitosis, and vascular co-option [47]. Vascular co-option is believed to be the main process responsible for the postoperative recurrence of GBM [6,48]. In fact, the success and speed with which GBM cells invade depends on their close contact with brain capillaries [49].

Several studies using different live microscopy approaches have shown that GBM cells use preexisting brain microvessels as scaffolding for migration [5,6,8,49]. Implanting cells from GBM cell lines into mouse brains is the most widespread strategy used to study GBM cell vessel co-option. When human GBM cells are cultured on mouse brain slices, they can convert normal capillaries into twisted vessels and produce thin, dynamic protrusions that can contact blood vessels [8]. In two-photon real-time imaging of grafted cells on brain slices, tumor cells developed a migratory morphology, angiotropic polarization, and co-opted blood vessels after 6 h [8]. Another study reported that the vascular-dependent spread of tumor cells started 24 h after in vivo implantation and continued throughout the course of the disease [6]. Co-option is more likely to occur along small capillaries, regardless of the type of vessel [50], and uses specialized cell structures to make contact [8]. Furthermore, the consequences of co-option include the displacement of normal astrocytic endfeet and that it enables tumor cells to assume control of vascular tone once the astrocytes are displaced from the vessel [50]. It has been reported that the regulator of G-protein signaling-5 (Rgs5) is expressed in PCs during physiological angiogenesis, but also during GBM–PC interactions in the tumor microenvironment [51]. Therefore, neural GBM cells go through immature developmental stages, as neural progenitors, to reactivate cell polarization mechanisms and angiotropism in order to interact with NVU cells and infiltrate normal, healthy parenchyma.

### 2.2. Flectopodia, the Execution Arm

**Figure 1:**
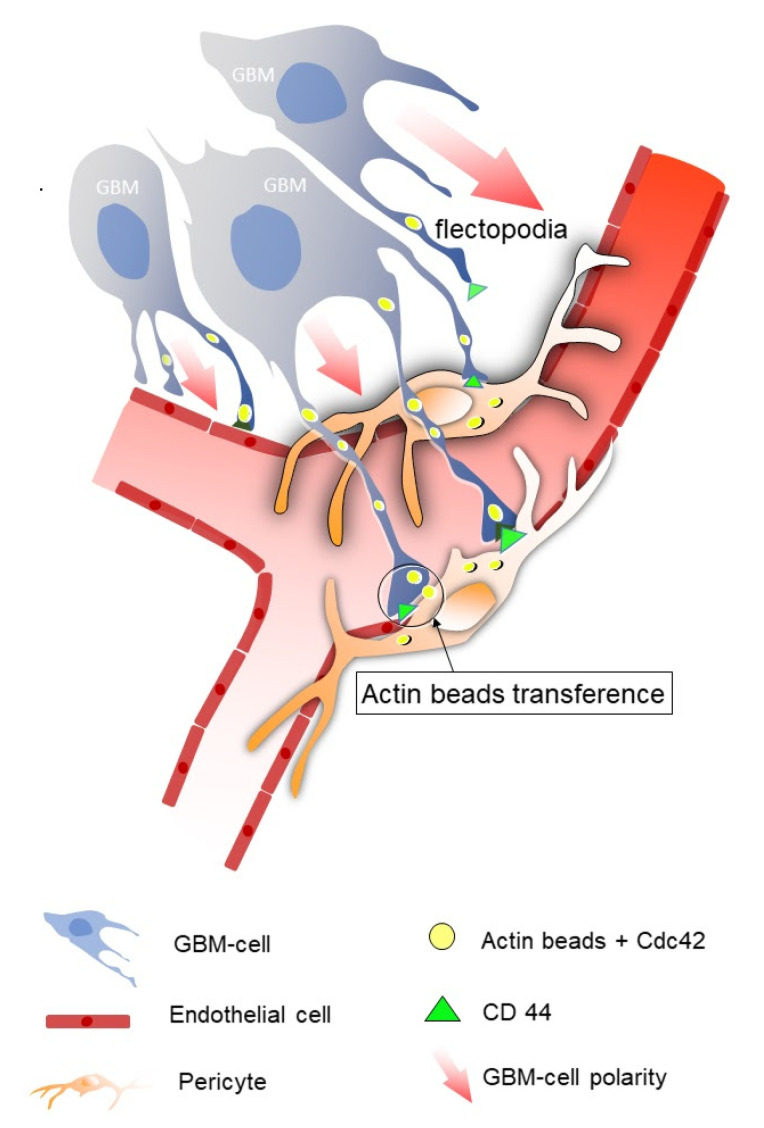
Diagram of the cell–cell interaction between GBM cells and pericytes. Tumor cells (gray) are shown interacting with blood vessels before co-opting them and modifying their contractility. The red arrows indicate tumor cell polarity. GBM cells develop flectopodia that contain actin beads which express Cdc42 (yellow ovals) and CD44 (green triangles) at the contact sites. The physical contact between the flectopodia and pericytes (orange) leads to the transfer of actin beads from tumor cells to the pericytes (yellow ovals within the pericytes).

During brain development, secretable morphogenetic signals that code positional information into developmental fields regulate the proliferation of cell progenitors and organ morphogenesis. The precision and temporal resolution of positional information during a specific timeframe requires contact-dependent signaling between cells. Cytonemes and tunneling nanotubes are specialized filopodia that are mainly composed of actin filaments which establish physical contact between cells [52,53]. Scaffold proteins are involved in the proximity and relative orientation of their molecular partners, and they are crucial for cell motility and polarity and intracellular signaling, among other functions. Interestingly, scaffold proteins have also been linked to cytoneme modulation; in fact, the Flot2 scaffold protein promotes Wnt3 transport in gastric cancer [54]. Other scaffold proteins have been detected in the GBM perivascular niche, which suggests that they may play a role in cytoneme organization [55]. Cytonemes are generated by stem cells to transfer polarized morphogenetic information to other cells during embryonic development (Figure 1). Therefore, the undifferentiated state of infiltrating glioblastoma cells favors the activation of polarization mechanisms towards NVU cells, where they mainly interact with PCs to co-opt vessels. When GBM cells are implanted in mouse slices and mouse brains, they start to produce thin, flexible dynamic filopodia, or cytoneme-like structures, polarized towards PCs around blood vessels. These cell specializations, first called flectopodia by Caspani et al. [34], have long extensions interrupted by cytoplasmic varicosities containing actin beads (Figure 1). Indeed, when GFP-actin-GBM cells were cultured with PCs, the PCs were found to contain cytoplasm from the tumor cells [8]. These findings show that tumor cell flectopodia are not only in physical contact with the surface of the PCs, but they can also transfer their cytoplasmic content to them (Figure 1). Active molecular and organelle transfer through cytoneme-mediated contacts between signal-producing and signal-receiving cells has also been described [52].

### 2.3. Consequences of GBM Cell–Pericyte Interaction during Co-Option

Cell–cell contact between tumor cells and pericytes changes the latter’s behavior. In vitro experiments on PCs cultured for 2 days on deformable silicone substrates, which were covered with human laminin to reproduce basal lamina, showed that the PCs generated compression forces around local nodes corresponding to areas with more contractile activity. Interestingly, when PCs were co-cultured with GBM cells, they started producing new wrinkles and destabilizing existing ones. These results show that GBM cells modify the contractile activity of pericytes, affecting the tone of co-opted vessels and subsequently modifying the vascular morphology, resulting in glomeruloid body formation at the GBM infiltrating edge [8]. Although vascular malformation in GBM has been associated with neovascularization, vascular co-option at the tumor infiltrating edge seems to be the trigger process of peritumoral glomeruloid bodies.

Furthermore, changes in the GBM extracellular matrix produce increased amounts of fibulins, among other substances, which are believed to facilitate GBM cell invasion. Studies suggest that PCs are one of the cells responsible for this change in extracellular matrix composition. In fact, the matricellular protein fibulin-7, which is crucial for the formation of aberrant blood vessels in GBM, was overexpressed in the glioblastoma microenvironment, particularly in perivascular cells such as pericytes [56].

The production of GBM–pericyte hybrids is another effect of the contact between tumor cells and PC. Notably, some of the hybrids were found on altered vessels and associated with oxidative/nitrative stress, indicating that the hypercontractility could be linked to the stress induced by the cancer cells. Caspani et al. proposed that fusion cells expressing nitrotyrosine could be a source of reactive oxygen and nitrogen species that corrupt pericyte contraction [8]. In fact, the reduction in oxidative stress revealed a decrease in PC immunologic conditioning by GBM cells [57].

Finally, the GBM cell–PC interaction produces important changes in the immune response of PCs, as will be explained later.

### 2.4. Molecules Involved in Vessel Co-Option in GBM

The molecules involved in GBM cell co-option were recently reviewed by Seano and Jain (2020) [47]. Some of the molecules identified are related to tumor cell chemotaxis (Bradykinin and CXCR4/SDF-1α), preventing vascular regression (angiopoietin-2 and VEGF), GBM proliferation and invasion (interleukin-8 (IL-8), MDGI/FABP3, and inositol-requiring enzyme (IRE)-1α), and the GBM cell–NVU cell interaction (CDC42 and ephrin-B2).

Cell division cycle 42 (Cdc42) GTPase regulates cell polarity across organisms, from yeast to humans, playing a central role in the morphogenesis of neuroepithelial cells. As Cdc42 regulates the actin–myosin cytoskeleton, it plays an important role in cell adhesion, vesicular trafficking, cell migration, and cytokinesis during embryonic development [58]. In fact, Cdc42 is associated with F-actin remodeling and GBM cell mobility. Furthermore, activation of the RXFP1-JAK3-STAT3-Cdc42 axis causes extensive filopodia formation [59]. The Cdc42 protein has been found in GBM cells and is a key molecule in vessel co-option and flectopodia function [8]. Studies have also shown that Cdc42 is involved in actin cytoskeleton organization [60] and the formation of cell protrusions, such as lamellipodia and filopodia [61]. Moreover, Cdc42 has been used as a target for antitumor drugs designed to prevent glioblastoma migration and invasion [62]. The Cdc42 enzyme is expressed in the flectopodia of GBM cells, co-localizing with the actin beads observed in the flectopodia varicosities. Interestingly, when Cdc42 was inhibited in tumor cells, there were fewer flectopodia, less vessel bending compared to the controls, and, most importantly of all, no vessel co-option [8].

Blood vessels also produce molecules that promote the interaction between tumor and vascular cells. Bradykinin is expressed in endothelial cells of co-opted vessels and is related to the chemotaxis of GBM cells and favors GBM invasion [63]. SDF-1α (expressed in blood vessels) and its receptor CXCR4 (expressed in GBM cells) are also associated with chemotaxis [64]. CD44 is believed to be another key molecule in the vascular co-option of GBM cells. CD44 is a transmembrane glycoprotein that is highly expressed in a lot of cancers, including GBM [65]. In a healthy brain, CD44 is involved in neuronal plasticity or development; however, in the context of cancer, it is related to tumor spread [65,66,67]. CD44 expression in GBM cells correlates with lower survival, greater tumor proliferation, increased treatment resistance, and more invasion [65]. In vitro experiments have demonstrated that knocking down CD44 increases the effects of Cdc42 inhibition, which suggests the two molecules have a synergistic effect [8]. On the other hand, CD99 is a transmembrane protein involved in normal cell adhesion and migration, but it is overexpressed in astrocytomas of varying degrees of malignancy. Interestingly, genes associated with filopodia formation are downregulated, which impairs cytoskeletal rearrangement and consequently inhibits tumor cell migration and invasion when CD99 is knocked out [59,68].

Flectopodia-dependent cell–cell interactions between GBM cells and PCs are also connected to the immune response, as explained later. Interestingly, when Cdc42 is inhibited in tumor cells, PCs transform into macrophage-like cells capable of phagocytizing the cancerous cells. This indicates another important role of the Cdc42 signaling pathway as it can be used to prevent PCs from transforming into macrophage-like cells and, subsequently, favoring tumor survival over its clearance [8].

## 3. Immunosuppressive Properties of Glioblastoma–Pericyte Interactions

Immune cells play a key role in host defenses against foreign antigens and unhealthy cells, including tumor cells. When they encounter signs of danger, immune cells are activated and modulate their immune functions. However, cancers have developed different strategies to suppress the antitumor immune response.

Studies indicate that glioma cells may interact with PCs, transferring malignant properties to and affecting the function of the latter [8,69]. In this situation, PCs do not stop tumor progression as their immune function fails to help eliminate GBM cells. It has recently been shown that direct interaction between pericytes and GBM cells (PC–GBM) is necessary to produce changes in the pericyte immune phenotype [8,33]. The pericyte–tumor cell interaction generated glioblastoma-conditioned pericytes (GBM-conditioned PCs). The GBM-conditioned PCs acquired an immunosuppressive phenotype that secreted high levels of anti-inflammatory cytokines, expressed immunosuppressive molecules, such as PDL-1, and reduced the expression of co-stimulatory molecules; this, in addition to a significantly impaired capacity to activate immunocompetent T cells, assisted tumor growth.

### 3.1. High Levels of Anti-Inflammatory Cytokines in Pericytes

The cytoneme-mediated interaction between PCs and GBM is known to produce in vitro changes in pericyte cytokine expression levels. The analysis of cytokines secreted from actin-GFP transgenic mice PCs (GFP-PC) co-cultured with a GBM human cell line revealed a significant increase in the production of anti-inflammatory cytokines IL-10 and TGF-β [33]. Moreover, in direct cell-to-cell interaction between PCs and GBM cells, the pericytes produce much lower levels of proinflammatory cytokines, such as IL-1, IL-23, IL-12, and TNF-α. These results indicate that the PC immunomodulatory phenotype can only be acquired in response to GBM cells when in the presence of direct cell-to-cell interaction, in this case, through the formation of stable flectopodia [35].

In vivo studies with grafts of co-cultured human GBM cells and GFP-PCs grafted into the brain cortex of an immunocompetent C57Bl/6 mouse model [70] have explored whether GBM-conditioned PCs also show an anti-inflammatory phenotype. In the brains of mice xenografted with GBM and GFP-PCs, the grafted PCs were found to express and secrete anti-inflammatory cytokines IL-10 and TGF-β [33,70]. The mechanisms modifying the expression of immunoactive molecules in conditioned PCs have not yet been determined, but they could be a consequence of changes in cell polarity due to transcription regulatory signals, including Cdc42 transfer from GBM cancer cells through cytoneme-like flectopodia [8].

### 3.2. The Expression of Immunosuppressive Membrane Molecules in Pericytes

Activated PCs present properties of macrophages, expressing macrophage markers and acquiring phagocytic activity [20,71]. An in vitro analysis of membrane molecules involved in the inhibition of antitumor responses, such as interleukin-1 receptor antagonist (IL-1Ra), showed that PCs express an immunosuppressive pattern of surface membrane molecules in response to interaction with GBM cells [33]. In humans, IL-1Ra is a protein encoded by the IL1RN gene. Upon interacting with GBM cells, pericytes respond by expressing high levels of IL-4Ra and IL-4RN mRNA [33].

**Figure 2:**
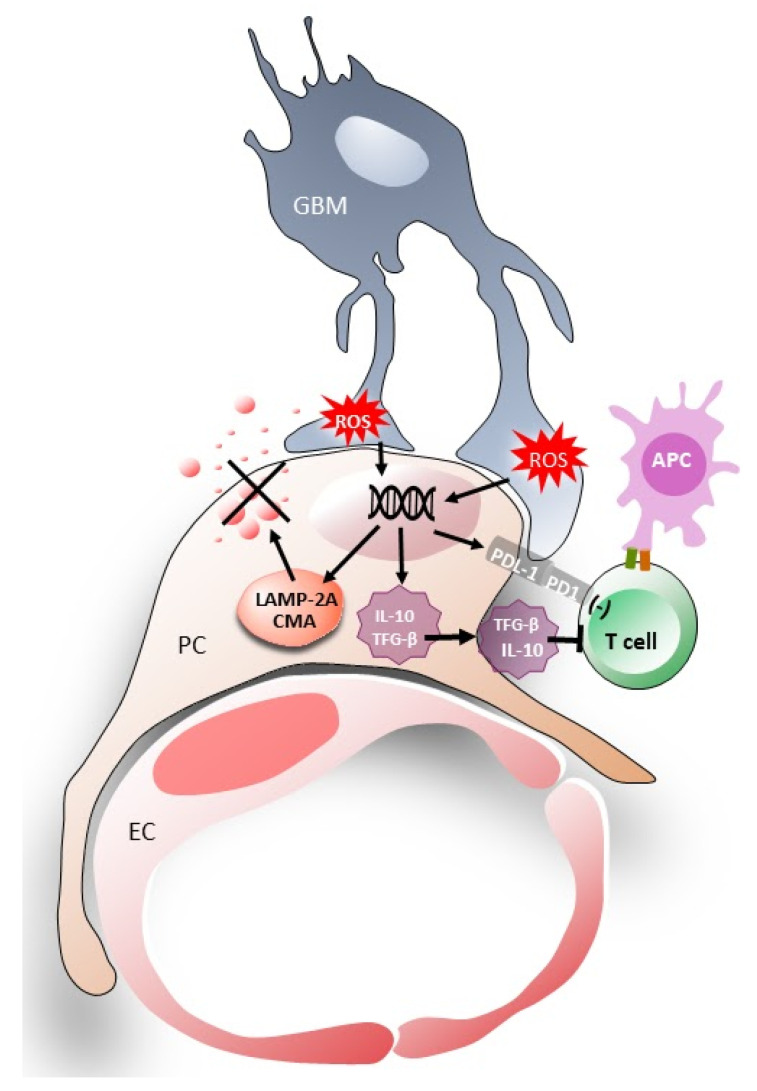
Diagram of the GBM–PC interaction inducing immunosuppressive properties in the PCs. A glioma cell (gray) is shown interacting with pericytes at which point it transfers malignant properties and affects PC function. The PC–GBM interaction increases the amount of ROS in GBM cells. This increase in ROS leads to: (1) upregulation of LAMP-2A and CMA, which increases the lysis of antitumoral proteins; (2) an increase in anti-inflammatory cytokine production and secretion (IL-10 and TGF-β); (3) the expression of immunosuppressive membrane molecules (PD-1); and (4) an impaired ability to activate T cells. APC: antigen-presenting cell; EC: endothelial cell; PC: pericyte; GBM: glioblastoma multiforme cell.

The immunosuppressive ligand of PD-1 (PD-L1) is a negative regulator of T cell activation and has been associated with glioblastoma progression [29,72,73]. Pericytes express PD-L1 in resting conditions, while its level of expression remained unchanged following in vitro and in vivo interaction with GBM cells [29,33,72,73] (Figure 2).

### 3.3. Reduced Expression of Co-Stimulatory Molecules and Inhibited T Cell Activation in Conditioned Pericytes

T cells are activated when their antigen-specific T cell receptor (TCR) interacts with a specific ligand. Effective T cell activation requires the engagement of two separate T cell receptors. The antigen-specific T cell receptor (TCR) binds foreign peptide antigen–MHC complexes, and the CD28 receptor binds to the B7 (CD80/CD86) co-stimulatory molecules expressed on the surface of antigen-presenting cells (APCs). In general, T cells do not recognize native protein antigen, but rather only antigen that has been physically altered (denatured or partially degraded) and subsequently presented in association with MHC class II (Ia) molecules by APCs. MHC II molecules are type I membrane glycoproteins that bind peptide fragments derived from exogenous protein sources, including viral and bacterial pathogens, and transport them to the cell surface for recognition by helper T cells. The immunogenic peptide derived from chicken ovalbumin, Ova323-339, has been used extensively to study the nature of MHC II–peptide binding and T cell activation [74]. Several studies have reported that PCs can present antigens to T cells regulating the activity of different T cells populations [23,28,75,76]. Immune synapses respond to specific cell-to-cell communication between T cells and PCs, which act as antigen-presenting cells in the brain [77,78]. Pericytes can present Ova323-339 peptide and activate CD4^+^ T cells [33]. A study with a human GBM cell line showed a significant reduction of CD80 and CD86 in pericytes co-cultured with GBM cells [33] and GBM-conditioned PCs showed a significantly impaired ability to activate T cells. CD4^+^ T cells were defective in proliferation and IL-2 cytokine production when co-cultured with antigen-loaded antigen-presenting cells in the presence of GBM-conditioned PCs [33].

Transcriptome modification in the expression of these molecules and/or alterations in the actomyosin cytoskeleton due to Cdc42 transfer may interfere with the formation of adequate immune synapses and, therefore, reduce the expression and clustering of MHC II molecules in conditioned pericytes. As is the case with leukemia cells, GBM cells may be able to modify actin cytoskeleton dynamics by increasing Cdc42 in pericyte cytoplasm, thus introducing adhesion and motility defects [79].

### 3.4. Pericytes Interacting with GBM Cells Promote Tumor Growth

GBM cell proliferation and enhanced tumor growth is facilitated by PCs in GBM cells co-cultured with pericytes [33]. GBM cell proliferation was studied in vivo using grafts of co-cultured human RFP-GBM cells and GFP-mouse PCs (GBM+PC) grafted onto the brain cortex of an immunocompetent C57Bl/6 mouse model [70]. The brains of mice with the GBM+PC xenograft presented a greater degree of perivascular infiltration of GBM cells [33].

Conversely, PCs did not increase proliferation in an in vitro model when co-cultured with GBM cells, which indicates that the underlying signal of cell proliferation was unidirectional and that cell specificity exists between GBM and PCs.

## 4. Induction of Chaperone-Mediated Autophagy (CMA) Activity in Pericytes

GBM cell interaction with PCs induces chaperone-mediated autophagy (CMA) in the PCs in response to the oxidative stress in GBM. CMA is a lysosomal process that selectively degrades intracellular proteins [80]. Oxidative stress-dependent signals from cancer cells modify specific protein degradation in PCs (Figure 2). The chaperone–substrate complex binds to lysosome-associated membrane protein type 2A (LAMP-2A), and the substrate protein then unfolds with the aid of chaperones. LAMP-2A acts as a transport channel in CMA substrate translocation. CMA activity depends directly on LAMP-2A levels at the lysosomal membrane. Regulation of CMA activity is critical to maintain cell function and homeostasis, selective degradation of proteins and to modulate their response to a wide variety of stimuli [81].

Although CMA and LAMP2A overexpression has been detected in several types of cancer [81], its implication in GBM has only just been observed in two recent studies, one of them in particular involved tumor-associated pericytes [57,82]. CMA is believed to regulate the function of some immune cells [83,84], including PCs [57,84]. Reactive oxygen species (ROS) are byproducts of the normal metabolism of oxygen and fulfill roles in cell signaling and homeostasis. ROS are present at low and stationary levels in normal cells. However, their levels can increase dramatically, potentially resulting in significant damage to cell structures, i.e., oxidative stress.

When GBM cells interact with PCs they produce more ROS which leads to an upregulation of CMA receptor expression (LAMP-2A) in PCs [84,85]. LAMP-2A is then delivered to the lysosomal membrane which causes abnormal upregulation of CMA activity in PCs [57]. Furthermore, this increase in LAMP-2A requires direct cell-to-cell interactions. Functional CMA activity in PCs is essential for the acquisition of the immunosuppressive function in response to the GBM interaction [57].

Other studies found that LAMP-2A upregulation protected GBM cells from apoptosis by degrading nuclear receptor co-repressor (N-CoR) and inhibiting the unfolded protein response (UPR) downstream [86].

A recent study reported that CMA plays a role in glioblastoma stem cells (GSCs). Proteomic and transcriptome analyses have revealed that CMA and LAMP-2A play an intrinsic role in maintaining GSC activity by modulating multiple pathways and processes [87].

The PC secretome comprises various functional molecules, including inflammatory modulators, angiogenic and trophic factors, and extracellular matrix proteins [88,89]. An increase in CMA activity in PCs is responsible for switching PC immune function and regulating properties associated with mesenchymal stem cells in those PCs. The co-culture of PCs with GBM cells increases the expression of several angiogenic factors, such as VEGF, angiotensin I, and cytokine IL-6, which are associated with changes in PC proliferation and regeneration [20,57,90].

Although the possible role of CMA in embryonic development has not been studied, alterations in macroautophagy have been associated with developmental abnormalities, such as neuronal migration, dendritic differentiation, and synapsis formation and pruning, which are often considered to be causes of autism spectrum disorder, tuberous sclerosis, and fragile X syndrome [91]. CMA and specific protein degradation may be essential processes in brain development. Moreover, the toxicity of the TDP-43 protein, a substrate of CMA in neural precursors, induces cell death [92].

### GBM-Induced CMA in Pericytes Helps Tumors Survive

This abnormal increase in CMA activity in PCs is responsible for the switch in PC immune function and for promoting more stable interactions with GBM, all of which increases tumor survival and prevents the secretion of proteins with antitumor activity [57]. GBM cells must induce CMA activity in PCs in order to stabilize the PC–GBM interactions that maintain interchange signals active through cytoneme-like flectopodia. In vitro, PC–GBM interaction reduces the expression of the interaction protein occludin through GBM-induced CMA, which suggests that the defective cell–cell interaction may be due to decreased occludin expression in PCs resulting from GBM-induced CMA. Occludin is vital for maintaining tight junctions between cells during vascular development and the integrity of the BBB, which suggests that a reduction in occludin may be involved in NVU functional alterations in gliomas [93].

CMA activity in PC is required to stabilize PC–GBM interactions, which help promote tumor survival. The co-culture of GBM with impaired CMA activity PCs (LAMP-2A knockout mice, KO PC) resulted in a higher percentage of GBM cell death and a significant loss of adherence in GBM [57]. Granulocyte-macrophage colony-stimulating factor (GM-CSF) is an important hematopoietic growth factor and immune modulator. GM-CSF also has a profound effect on the functional activities of various circulating leukocytes. It is produced by various types of cells, including T cells, macrophages, endothelial cells, and fibroblasts, upon receiving immune stimuli [94]. The co-culture of GBM with CMA-deficient PCs increased GM-CSF secretion from the GBM cells, which consequently reduced tumor cell survival and inhibited GBM–PC interactions [57].

## 5. Changes in the Microvesicular and Protein Antitumor Secretome

The physical interaction between GBM cells and PCs can produce GBM–pericyte hybrids, which alters pericyte contractility, prevents pericytes from transforming into macrophage-like cells, and inhibits the inflammatory response [8,33,57,95]. Altogether, this favors tumor progression by promoting its nutrition and invasion through co-option, and tumor survival, by establishing immunotolerance. However, the cell–cell contact necessary for tumor progression must induce subcellular changes in the perivascular cells that underlie the transformation from healthy to corrupted pericytes.

As explained above, PCs develop immunosuppressive characteristics after coming into contact with GBM cells. Tumor cells induce an abnormal increase in CMA in pericytes that acquire an anti-inflammatory phenotype, thus inactivating the T cell response [33,57]. The observation that CMA-deficient PCs prevented GBM cell-induced transformation was explained by the fact that the study’s pericytes had different levels of gene/protein expression than the control’s pericytes [96].

### 5.1. Pericyte Secretome

Pericytes are located in a strategic position between blood vessels and the surrounding tissue, so they are the first cells to sense environmental changes. They respond to environmental stimuli by secreting molecules that act over both short and long distances [97]. In control cultures, brain PCs secrete immune mediators, such as cytokines, chemokines, nitric oxide [21], major histocompatibility complex (MHC) proteins [23], adhesion molecules [98], and angiogenic and neurotrophic factors [99,100], all of which would play a fundamental role in the development and maturation of the brain and NVU. When PCs are exposed to certain stimuli, they begin to release new molecules, enriching the “basal secretome” and converting it into an “induced secretome” that depends on the nature of the inducer and the origin of the pericytes [97].

Cells can secrete molecules directly or through microvesicles, small membrane-enclosed vesicles that detach from the plasma membrane [101]. Human brain PCs release microvesicles under specific conditions. In vitro studies have shown that when PCs are stimulated with lipopolysaccharide, a stimulator of the innate immune system, they begin to release microvesicles with high amounts of cytokines. Similarly, when the inducer is platelet-derived growth factor-BB (PDGF-BB), the PCs release more microvesicles containing even greater amounts of growth factors [90,97].

Pericytes are involved in multiple processes through their secretome. The in vitro study of pericyte secretome showed that pericytes are vital for tumor immune response, inflammatory reaction, and immune evasion [97].

### 5.2. Secretome of Pericytes in Tumor Conditions

As explained previously, pericytes are known to assist tumors in tumor cell infiltration and immune evasion, which are the main obstacles in GBM treatment. In addition to changing membrane expression proteins, GBM-contacted pericytes secrete a group of factors that help suppress the immune response [102]. They also secrete some interleukins responsible for metastasis and, therefore, tumor survival [27,33]. Furthermore, a molecular transcriptome study of the glioblastoma perivascular niche in humans compared glioblastoma vascular cells (GVCs) with normal vascular cells and found the former exhibited over 400 GVC-enriched genes known to regulate GBM-perivascular interaction. A significant enrichment in glioma-related proteins, invasiveness, and proliferation, and a negative association with tumor necrosis and cell death was also observed in GVCs. Integrin-binding sialoprotein (IBSP) isolated from the transcriptome analysis was put forward as a mediator of tumor cell migration [103].

The interaction between tumor cells and PCs changes the pericyte secretome to an immunotolerant secretome rich in factors that impair the immune response and tumor clearance. This reaction is apparently due to an increase in CMA induced by tumor cell–pericyte contact. In fact, autophagy-ablated pericytes do not suppress immune function [96].

### 5.3. CMA-Induced Changes in Pericyte Transcriptome Profiling/Secretome

Pericytes are considered the first line of defense against GBM because they are associated with the vasculature, they act as macrophages, and they can present antigens, which initiates a proinflammatory response [23,71,104]. However, this response is blocked when GBM induces CMA in PCs and recovered when CMA is impaired [57]. Given that CMA depends directly on LAMP-2A levels at the lysosomal membrane [105], pericytes isolated from brains of Lamp-2a^-/-^ mice were used as autophagy-ablated pericytes. Although these PCs contacted GBM cells, they did not increase CMA activity, and the immune and inflammatory response pathways were found to be upregulated, as was the phagosome pathway. Interestingly, genes related to cell adhesion molecules (CAMs) were also upregulated in autophagy-ablated pericytes [96]. CAMs, which are involved in cell adhesion to the substratum and extracellular matrix, have been linked to cancer [106]. CAMs were differentially regulated in morphogenesis and cell migration during tumor development and cancer progression [107].

Genes related to angiogenesis, actin and adherens junctions, on the other hand, were downregulated in autophagy-ablated PCs, which is consistent with the pericytes’ antitumoral behavior observed previously when CMA is blocked [57,96].

In one of those studies, Molina et al. analyzed the differential expression of protein secretion (secretome) from GBM and control or autophagy-ablated pericyte co-cultures [96]. They found that several protumor proteins were overexpressed in the control PC–GBM co-cultures compared with autophagy-ablated PC–GBM co-cultures, namely cell adhesion, actin cytoskeleton regulation, and angiogenic proteins. Cell adhesion and actin cytoskeleton proteins are involved in directional sensing, cytoskeletal dynamics, cell–cell junction assembly/disassembly, and integrin-matrix adhesion, which are essential processes in cell migration [108]. Moreover, the actin cytoskeleton is crucial to the formation of a specialized type of cell–cell junction between the immune cell and its prospective target, called the immunological synapse (IS). ISs perform different functions, such as cytokine secretion and regulating lymphocyte activation and maturation [79]. Absi et al. proposed that the actin cytoskeleton in cancer cells structures itself to escape the immune response [109]. Moreover, gene expression profiling of cytotoxic T lymphocytes collected from patients with lymphocytic leukemia revealed alterations to the proteins involved in actin cytoskeleton regulation due to the presence of inhibitory ligands on leukemia cells. In this case, actin cytoskeleton dysregulation resulted in adhesion and motility defects [110,111]. Interestingly, ISs are also formed between cytotoxic lymphocytes (T cells) and antigen-presenting cells (APCs), stimulating immune responses on both sides of the IS. Increased amounts of inhibitory ligands in cancer cause actin to accumulate around the ISs, blocking correct immune function. These ISs between cancer cells and T cells have been called evasion synapses [79]. As explained earlier, PCs can present antigens on MHC proteins to T cells regulating their activity. The differential expression of actin cytoskeleton regulators together with the Cdc42 transfer observed in GBM–PC contacts may result in actin cytoskeleton dysregulation, thus producing evasion synapses that prevent the immune response in GBM.

On the other hand, an analysis of the secretome of autophagy-ablated PCs cultured with GBM cells demonstrated that actin cytoskeleton regulators were not differentially expressed [96]. However, the authors found that 15 antitumor proteins were differentially expressed, including proteins that inhibit interaction with tumor cells, secreted proteins associated with tumor apoptosis, and anti-angiogenetic proteins.

Secretome analysis reveals that cell–cell contact is a key element in promoting tumor progression through angiogenesis, co-option, and by generating an immunosuppressed microenvironment that induces abnormal CMA in pericytes. This subsequently causes changes in the actin cytoskeleton that probably affect pericyte–T cell interactions.

## 6. Future Strategies and Routes for Targeting GBM–Pericyte Interactions

The physical interactions mediated by cell protrusions (filopodia or flectopodia) are fundamental for understanding the intercellular communication processes underlying cell polarity. These interactions are an essential component of embryonic development processes (cell proliferation and migration) and tumor growth (cell infiltration and vascular co-option). The flectopodia-mediated physical interaction between GBM cells and PCs, that is, perivascular cells in the tumor microenvironment, produces important functional changes in the PCs’ response to tumor cells. Specifically, these are vascular co-option, which mediates vascular malformation, and PC transcriptome, which modifies the PC secretome and leads to the development of an immunosuppressive phenotype that promotes tumor immune tolerance.

Due to the important role of tumor cell–pericyte contact in the progression of GBM, some authors have proposed PCs as therapeutic targets for different approaches, such as tracking tumor progression by radioactively labeling PCs, inhibiting pericyte proliferation, and blocking pericyte–GBM cell interactions via Cdc42 inhibition [112]. Targeting PCs could represent an interesting therapeutic strategy for GBM. However, healthy PCs are a powerful resource in the immune response that must be protected to promote tumor control in GBM. In fact, it has been shown that the additional targeting of pericytes in subcutaneous tumors did not increase the efficacy of any other antitumor treatments [113]. Other authors propose the use of tyrosine kinase inhibitors, such as ibrutinib and sunitinib, which selectively and exclusively disrupt glioblastoma-derived pericytes [114]. This approach would target only some of the PCs while preserving a significant population of healthy PCs. In vitro, however, these drugs seem to kill healthy pericytes [115], which could impact in vivo brain function. Furthermore, non-glioblastoma-derived PCs can also be corrupted by contact with GBM cells. Therefore, therapeutic strategies targeting GBM–pericyte contacts could represent a more effective antitumoral approach. As explained previously, Cdc42 is required for co-option and flectopodia formation. Consequently, inhibiting Cdc42 activation [116] or blocking *Cdc42* gene production, e.g., the Cdc42 GTPases, both represent interesting strategies for preventing PC–GBM cell contact and PC corruption. ARN22089 is a novel compound that has been reported to block the interaction between Cdc42 GTPases and their effectors in mouse melanoma models and in patient-derived xenografts in vivo [117]. This last strategy could alleviate some of the hematological side effects reported with the use of Cdc42 activation inhibitors.

We have shown that the increase in CMA in GBM-conditioned PCs underlies PC transformation and flectopodia stabilization, which suggests that CMA regulation could be another therapeutic target in peritumoral PCs. While CMA activity is greater in cancer cells and pericytes, it would seem the anti- or pro-cancer function of CMA depends on GBM cell transformation and PC expression. This highlights the importance of more research into context-dependent therapy [118].

These advances hold a lot of promise for the future treatment of GBM. However, our knowledge of PC–GBM cell interactions is limited, and further studies are needed to fully understand the molecular mechanisms underlying GBM–PC communication and the changes in PC behavior so that we may move closer to a definitive treatment for this devastating disease.

## Data Availability

Not applicable.
